# Development and Validation of the First Iranian Questionnaire to Assess Quality of Life in Patients With Heart Failure: IHF-QoL

**DOI:** 10.5812/cardiovascmed.4186

**Published:** 2012-11-01

**Authors:** Nasim Naderi, Hooman Bakhshandeh, Ahmad Amin, Sepideh Taghavi, Masoumeh Dadashi, Majid Maleki

**Affiliations:** 1 Cardiac Electrophysiology Research Center, Rajaie Cardiovascular Medical and Research Center, Tehran University of Medical Sciences, Tehran, IR Iran; 2 Cardiovascular Intervention Research Center, Rajaie Cardiovascular Medical and Research Center, Tehran University of Medical Sciences, Tehran, IR Iran; 3 Department of Heart Failure and Transplantation, Rajaie Cardiovascular Medical and Research Center, Tehran University of Medical Sciences, Tehran, IR Iran; 4 Echocardiography Research Center, Rajaie Cardiovascular Medical and Research Center, Tehran University of Medical Sciences, Tehran, IR Iran

**Keywords:** Quality of life, Heart failure, Validation studies, Iran

## Abstract

**Background:**

In its Constitution of 1948, WHO defined health as “a state of complete physical, mental, and social well-being, and not merely the absence of disease and infirmity” . In 1994, the Agency for Health Care Policy and Research published clinical practice guidelines recommending providers to routinely evaluate patients’ HRQoL (Health Related Quality of Life) and use their assessment to modify and guide patient care.

**Objectives:**

to create a valid, sensitive, disease-specific Persian health status quality of life questionnaire for patients with chronic heart failure in Iran.

**Materials and Methods:**

Considering the existing relevant inventories and scientific literature, the authors designed the first draft of questionnaire which was modified and validated, using expert opinions and finalized in a session of expert panel. The questionnaire was processed among 130 patients with heart failure. Construct validity evaluated by principle component factor analysis, and promax method was used for factor rotation. MacNew quality of life questionnaire was selected to assess convergence validity, and the agreements were measured in 60 patients. Discriminant validity was also assessed. Thirty patients were followed for 3 months and responsiveness of questionnaire was measured. Cronbach's alpha, item analysis, and Intra-class correlation coefficients (ICCs) were used to investigate reliability of questionnaire. SPSS 15 for Windows was applied for statistical analysis.

**Results:**

Principle component factor analysis revealed 4 main components. Sub-group analysis suggested that IHF-QoL questionnaire demonstrated an acceptable discriminant validity. High conformity between this inventory and MacNew questionnaire revealed an appropriate convergence validity. Cronbach's alpha (α) for the overall questionnaire was equal to 0.922. Intra-class correlation coefficients (ICCs) for all components were significant (from. 708 to. 883; all P values < 0.001). Patients fallow-up revealed an acceptable responsiveness of our questionnaire.

**Conclusions:**

IHF-QoL questionnaire is a valid and reliable inventory. It can be applied in daily clinical practice and in the clinical research context.

## 1. Background

In its Constitution of 1948, WHO defined health as “a state of complete physical, mental, and social well-being, and not merely the absence of disease and infirmity”. In 1994, the Agency for Health Care Policy and Research published clinical practice guidelines recommending providers to routinely evaluate patients’ HRQoL (Health Related Quality of Life) and use their assessment to modify and guide patient care (1). Quality of Life is considered as the most important concept in all medical illnesses that involves all relevant factors to health status directly and indirectly. Many medical interventions are designed to improve quality of life rather than prolong the life; this obliges a mandatory standard measurement for quality of life. Although a number of quality-of-life instruments have been developed for general population, they are unlikely to detect small and clinically important changes. Therefore, investigators have developed disease-specific instruments for patients with cancer, joint disease, heart disease, and chronic lung disease (2). Different illnesses may affect different organ functions and lead to different physical and emotional problems (2).

Chronic heart failure is a chronic condition with enormous impact on the lifestyle of patients (2-11). In Iran the prevalence of chronic heart failure has been estimated at the rate of 3337 in 10000 (9). Heart failure adversely affects quality of life because of changes in lungs, circulation, and skeletal muscle which often results in recurrent hospital admissions due to symptoms of breathlessness, peripheral edema, and overwhelming fatigue, all of which interfere with day-to-day activities and impose massive limitations on occupational and recreational activities (7, 8, 11). These limitations, from a patient’s point of view, are superior, yet more important than famous symptoms and signs being addressed and treated by most physicians. Traditionally, physicians apply New York Heart Association (NYHA) classification (a combination of physical limitations and symptoms) to assess functional status of patients. However , this simple system is subject to inter-observer inconsistency, shows only a limited range of health status, and is applied from a physician’s perspective instead of the patient’s (5).

Many generic instruments such as Short-Form 36, Short-Form 12, and Euroqol as well as disease-specific tools such as MacNew (6), Minnesota Living with Heart Failure, and Kansas City Cardiomyopathy (5) questionnaires have been developed to measure the quality of life. MacNew has been designed to measure quality of life in patients with a heart problem (6). Other available disease-specific HRQoL instruments for heart failure include Minnesota Living with Heart Failure Questionnaire and Kansas City Cardiomyopathy Questionnaire (KCCQ) which are currently employed as the most standard QoL assessment tools applied in most clinical trials conducting in heart failure field (5). Kansas City Cardiomyopathy Questionnaire (KCCQ) is a self-processed 23-item questionnaire developed to provide a better description of HRQoL in patients with heart failure compared to Minnesota Living with Heart Failure Questionnaire (5).These specific questionnaires allow the measurement of significant clinical domains, and are sensitive to identify clinical changes. However, because these tests evaluate domains that also reflect a patient’s cultural and ethnic background and are generally worded using idiomatic expressions typical of one’s own language and environment, any HRQoL questionnaire should be validated after translation to other languages, a complex and costly procedure (1-6, 9, 10). Currently, there is no standardized, comprehensive, and regionally accepted disease specific HRQoL instrument for CHF in Iran. Designing a new disease and language specific instrument could be another way to get a valid QoL questionnaire.

## 2. Objectives

The main objective of this study was rendered to create a valid, sensitive, disease-specific Persian health status measuring tool for patients with chronic heart failure in Iran.

## 3. Materials and Methods

### 3.1. Development of Questionnaire

The development of Iranian Heart Failure Quality of Life (IHF-QoL) questionnaire was begun by reviewing the existing literature and currently available health related QoL instruments (general measures including WHOQOL, and heart failure disease specific including Minnesota Living with heart failure questionnaire and Kansas City cardiomyopathy questionnaire) (5). After that, the authors designed the first draft of inventory. The questions addressed the concepts in patients' symptoms, physical activity, psychosocial life, and self-care. All the questions (items) were designed as 3 or 4 point Likert scale response.

Content validity and face validity of the questionnaire items were examined by sub-specialty experts of cardiovascular medicine and research (n = 20). The experts reviewed the questions and determined the degrees of their relevancy in a three point scale response (non-relevant, moderately relevant, and relevant). They also expressed their opinions about suitability of each question. Designers of the questionnaire, then, reviewed the suggestions and revised the questionnaire, accordingly. This inventory was processed as a pretest over a sample of 20 volunteers of patients examining their fluency and operability. Then, some relevant schematic drawings were added to question number 7(physical limitation) to get better clarified. The newly designed inventory was examined on 45 patients. After final revisions, 15 questions were prepared to test for validity and reliability (Appendix) in the following domains:

1. Symptoms and their severity (questions 1,2,3,4, and 6);
2. Physical limitations, considering METS chart of daily living activities accompanied by relevant drawings for better clarification of the questions(questions 7-1 to 7-6);
3. Social interference (questions 8, 10, 12, and 13);
4. Psychological condition (questions 5, 9, and 11);
5. Self-efficacy and knowledge (questions 14 and 15).

Question No. 16 was added to the inventory as a "conclusive item". In this question, patients were asked to score their own overall quality of life as: unfavorable, moderately favorable, and favorable. This item considered as a controlling measure and its correlation with different components of questionnaire was assessed. Questions related to patients' demographic and background data were also added. Finally, in a discussion session held by participating 7 experts as focus group, the IHF-QoL questionnaire was approved.

### 3.2. Study Population

The study protocol was approved by local Ethics Committee. The patients with documented heart failure who were referred to Heart Failure Clinic of Rajaie Cardiovascular Medical and Research, were12-year-old and over and capable of answering the questionnaire were enrolled. Filling the form was helped by Clinic nurse or patient’s relatives if patient was unable to read or write. Patients were all assessed by a heart failure specialist and clinical status assessment was done simultaneously. All patients had a clinical diagnosis of heart failure with either reduced or normal ejection fraction (including patients with predominant right sided heart failure)

### 3.3. Convergent Validity

To assess the degrees of similarities between our inventory and other existing questionnaires, we selected MacNew cardiovascular health-related quality of life questionnaire, which had been adapted and validated for Persian language by Asadi Lari et al. in 2003 (12).Twenty eight patients, who were interested in participation, were asked to answer the MacNew HR-QoL questionnaire after completing our questionnaire. 

### 3.4. Patients’ Follow-up

We planned to follow the patients for 3 months to assess the responsiveness of the questionnaire according to the changes in patients’ clinical condition. At the time of result reporting, 19 patients with at least one level improvement in their NYHA functional class status had a complete follow-up period. Questionnaire scores were computed and compared, twice.

### 3.5. Statistical Analysis

Exploring the construct of the inventory was performed using principle component factor analysis (PCA). Eigenvalue more than 1 (Kaiser’s criterion) was considered to determine the main components. Promax method with Kaiser Normalization was used for rotation of retained components. Factor loadings ≥ 0.5 was considered as significant and were entered in the final questionnaires. Cronbach's alpha was calculated to determine internal consistency of items. Also, intra-class correlation coefficient was used to show consistency and reliability of questionnaire. For more investigation about validity, sub-group analysis was performed. Data described as mean ± standard deviation or median (inter-quartile range) for interval variables, and count (percent) for categorical variables. One sample Kolmogorov-Smirnov test was applied to investigate normal distribution for interval variables. Comparisons between subgroups were carried out by using Student's t-test and one-way analysis of variance models with Bonferroni post-hoc test. Pitman's test was used to investigate difference between variances of QoL scores resulted from our questionnaire and that of MacNew. Pearson's r and Spearman's rho were also used to show the correlations between variables. Comparison between questionnaire scores, before and after the treatment, was carried out by paired t test. P <. 05 considered as statistically significant. Agreement between two questionnaires was investigated by using a Bland-Altman plot (13). Statistical analysis was performed by using SPSS for Windows version 15 (SPSS Corporation, Chicago, Illinois). 

## 4. Results

One-hundred and thirteen patients (mean age = 50 ± 18.3 years) participated. Women/men ratio was 36/77. The majority of patients were in NYHA function class II (35.4%) and III (38.1%). Other Patients’ background data is shown in Table 3. 

**Table 1.** Factor Analysis on Iranian Heart Failure Quality of Life (IHF-QoL) Questionnaire

	Description	Rotated Factor Loadings Matrix^b^			
		Factor 1	Factor 2	Factor 3	Factor 4
SHF^a^-1	Dyspnea	0.774			
SHF-2	Edema of Lower Extremities	0.659			
SHF-3	Weakness and Fatigue	0.865			
SE-8	Limitation in Social Activities	0.781			
PSY-9	Sexual Activities	0.724			
SE^a^ -10	Dependency in Personal Activities	0.817			
PSY-11	Anxiety/ Depression Symptoms	0.762			
SE-12	Economic Burden	0.544			
SE-13	Burden to Family	0.694			
SHF-4	Palpitation		0.609		
PSY^a^ -5	Quality of Sleep		0.833		
SHF-6	Gastrointestinal Symptoms		0.787		
DA^a^ -7	Limitation in Personal Daily Activities				
DA-7 (1)	Light Activities			0.693	
DA-7 (2)	Light to Moderate Activities			0.776	
DA-7 (3)	Moderate Activities			0.872	
DA-7 (4)	Moderate to Heavy Activities			0.903	
DA-7 (5)	Relatively Heavy Activities			0.848	
DA-7 (6)	Very Heavy Activities			0.730	
SC^a^ -14	Knowing the Aggravating/ Alleviating Factors				0.857
SC-15	Remembering the Symptoms Leading to Visit Doctor				0.895
Total variance, %		23.3	11.8	23	9.3

^a^ Abbreviations: DA, personal daily activities; PSY, psychological aspect; SC, self-care aspect; SE, socio-economic aspect; SHF, symptoms of heart failure

^b^ Extraction Method: Principal Component Analysis; Rotation Method: Promax with Kaiser Normalization

### 4.1. Investigating the Construct Validity: Factor Analysis

Principle component factor analysis was employed to investigate structure of inventories. Kaiser’s criterion (Eigenvalue = 1.0) was considered to determine main factors by which 4 components were found that explained 67.4% of the common variance shared by 15 items in our QoL questionnaire. Bartlett's test of sphericity was significant (χ^2^ = 844.4, df = 190; P <0.001). Kaiser-Meyer-Olkin measure of sampling adequacy (KMO) was 0.875, proposed that the degree of common variance among the 15 items was “meritorious”. In order to optimize interpretation, a Promax rotation was performed. The results are presented in Table 1. First column of Table 1 indicated the name of items, which consisted of original category of each question (showed by 2-3 letters acronyms) and the number of question in the questionnaire. All the factor loadings were in acceptable range (0.609 to 0.903). It was clarified that the inventory was structured by four components. The first component included nine items belonged to original categories of symptoms, and socio-economic and psychological aspects of quality of life. These items referred to symptoms or impaired activities interfering with the patient’s social life. The second component consisted of three items presented the symptoms which affect more the patients’ personal feelings. Two other components are the same as original categories of questionnaire; component 3 represented the limitations in patients’ daily activities and component 4 showed their self-care.

**Table 2.** Reliability Statistics of Iranian Heart Failure Quality of Life (IHF-QoL) Questionnaire

Component/Items	Cronbach's Alpha	Cronbach's Alpha if Item Deleted	Corrected Item- Total Correlation	ICC^a^(CI95, %)
Factor 1	0.875			0.875^b^(0.830-0.913)
Dyspnea		0.859	0.658	
Edema of Lower Extremities		0.868	0.578	
Weakness and Fatigue		0.848	0.760	
Limitation in Social Activities		0.854	0.733	
Sexual Activities		0.864	0.591	
Dependency in Personal Activities		0.855	0.726	
Anxiety/ Depression Symptoms		0.861	0.632	
Economic Burden		0.877	0.425	
Burden to Family		0.871	0.507	
Factor 2	0.708			0.708^b^(0.624-0.747)
Palpitation		0.691	0.680	
Quality of Sleep		0.694	0.570	
Gastrointestinal Symptoms		0.629	0.669	
Factor 3	0.883			0.883^b^(0.843-0.916)
Limitation in Light Activities		0.878	0.621	
Limitation in Light to Moderate Activities		0.855	0.749	
Limitation in Moderate Activities		0.841	0.818	
Limitation in Moderate to Heavy Activities		0.844	0.809	
Limitation in Relatively Heavy Activities		0.863	0.700	
Limitation in Very Heavy Activities		0.889	0.550	
Factor 4	0.741			0.741^b^(0.625-0.822)
Knowing the Aggravating/ Alleviating Factors		-	0.588	
Remembering the Symptoms Leading to Visit Doctor		-	0.588	

^a^ ICC: Intra-class Correlation

^b^ *P* value < 0.001

### 4.2. Reliability and Item Analysis

Cronbach's alpha (α) for the overall questionnaire was equal to 0.922. Also, α was computed for each component to determine internal consistencies. As shown in Table 2, α was acceptable for all components (from 0.708 to 0.883). Cronbach's alpha if item deleted was also computed (Table 2). The results showed that by item deletion no important improvement occurred in α that meant we could keep all items. The range of α for the first two components was equal to 0.580 to 0.680. In the last component, α fell in a range of 0.070 – 0.349that was a weak result. The results of corrected item to total correlations (ITCs) are presented in Table 2. Mean ± SD of ITCs was equal to 65 ± 10, which indicated appropriate correlations between the items. Intra-class correlation coefficients (ICCs) for all components were significant (from 0.708 to 0.883; all P values < 0.001). Other Patients’ background data is shown in Table 3.

**Table 3.** Quality of Life Score in Different Sub-Groups

	Mean QOL Score ± SD	*P* value
Sex		0.453
Female (n = 36)	38.6 ± 10.2	
Male (n = 77)	40.7 ± 9.6	
Marital Status		0.827
Single/Widow (n = 24)	39.6 ± 10.4	
Married (n = 89)	40.1 ± 9.7	
Education Status		0.010
Primary School (n = 45)	36.8 ± 8.0^a^	
High School (n = 52)	41.7 ± 10.0^a^	
Academic Education (n = 16)	43.9 ± 11.5^a^	
Socioeconomic Status		0.397
High (n = 20)	42.8 ± 7.8	
Middle (n = 58)	40.6 ± 10.2	
Low (n = 30)	38.9 ± 9.8	
Regular Exercising		0.312
No (n = 61)	37.9 ± 10.2	
Yes (n = 29)	40.1 ± 7.5	
NYHA Function Class		< 0.001
Class IV (n = 9)	26.8 ± 5.0^a^	
Class III(n = 40)	35.2 ± 6.7^a^	
Class II (n = 43)	43.9 ± 7.2^a^	
Class I (n = 21)	47 ± 11.2^a^	
Full Medical Treatment		0.017
No (n = 38)	36.9 ± 10.2	
Yes (n = 75)	41.6 ± 9.3	
Stability of Clinical Situation		0.007
No (n = 15)	33.7 ± 8.4	
Yes (n = 98)	41 ± 9.7	
Outpatient Visit		0.026
No (n = 86)	41.2 ± 9.9	
≥ 1/previous month (n = 27)	36.4 ± 8.7	
Hospital Admission		0.001
No (n = 88)	41.7 ± 9.8	
≥ 1/previous month (n = 25)	34.2 ± 7.6	
Emergency Room Admission		< 0.001
No (n = 84)	42.1 ± 9.6	
≥ 1/tprevious month(n = 29)	33.8 ± 7.6	

^a^ Significant difference in pairwise comparisons, using Bonferroni posthoc test; P values < 0.05

### 4.3. Convergent Validity

**Figure 1.**
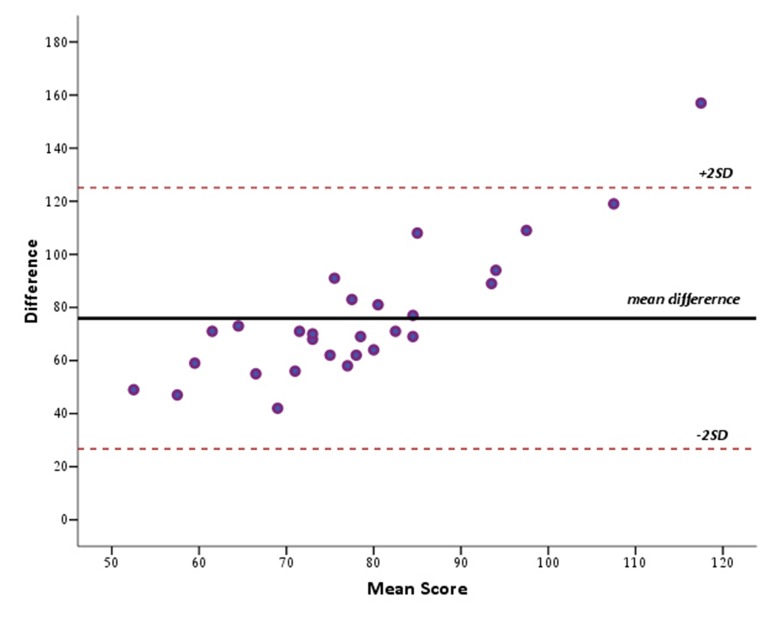
Bland-Altman Plot for Assessing the Agreement Between IHF-QOL and Mac-New HR-QOL Questionnaires

Twenty eight patients were asked to answer MacNew quality of life questionnaire at the same session they answered IHF-QoL questionnaire. The conformity between the scores of two questionnaires was assessed by Bland-Altman plot (Figure 1) and Pitman's test. Only one measurement was out of the acceptable range. P value for the equality of the variances was equal to 0.323 which proposed the equality of results. These findings showed that the conformity between two inventories was appropriate. It can be concluded that the results of IHF-QoL was concordant with MacNew questionnaire and accordingly, convergent validity of the questionnaire could be confirmed.

### 4.4. Quality of Life Score in Iranian Heart Failure Patients

Maximum score of IHF-QOL was equal to 66. Median score (Inter-quartile range) of questionnaire was equal to 41(32 – 47) which proposed a relatively moderate satisfaction among participants. Most of the patients’ dissatisfaction could be judged by limitations in their social and daily activities. Spearman’s correlation coefficient (ρ) between IHF-QoL score and “conclusive item” in the questionnaire (question 16) was equal to 0.548 (P < 0.001). Mean ρ between each component and “conclusive item” was equal to 0.378. All components (except component 4) and total scores had significant correlations with the conclusive item. This showed an acceptable reliability of questionnaire.

### 4.5. Discriminant Validity

Discriminant validity was shown by comparison QoL scores between different sub-groups of patients. It was expected that patients in better conditions achieve higher scores. The results of sub-group analysis are presented in Table 3. As noted in Table 3, patients in lower NYHA function classes, taking full medical treatment and showing stable clinical conditions and those who did not need to admit had significantly higher scores compared to other patients, referred to as their better quality of life. A negative, weak association was found between IHF-QoL score and patients' age (Pearson's r = -0.225; P = 0.017). Also, patients who had educated up to primary school level had lower scores than those with high school grades or academic educations. On the other hand, these differences were not observed between sub-groups which didn't have any expected effect on patients' quality of life (sex, marital status). The above-mentioned results proposed that IHF-QoL questionnaire achieved an acceptable discriminant validity.

### 4.6. Responsiveness

Three months after treatment, 30 patients were visited and asked to answer the form. The patients’ clinical condition were assessed by cardiologists blinded to questionnaire results.Nineteen patients had an improvement in their clinical condition (at least one class improvement in their NYHA function class score). Comparing the scores of different components and total score of IHF-QoL questionnaire before and after the treatment showed that the increase of scores in patients, parallel to the improvement of their health states, was significant (all p values were < 0.05). Thereby, the sensitivity of questionnaire to important changes was acceptable. The rest of patients had no changes or showed worsening in their NYHA function class (nine and two patients, respectively).

## 5. Discussion

Over the past two decades, there has been growing interest in assessing the Health related QoL of patients with heart failure, especially for measuring outcomes in health services research and clinical trials. Quantifying the impact of new treatments of heart failure on patients, their survival, their symptoms, and their QoL is very important for physicians to monitor and improve quality of cares. This study reports designing and subsequent validation of a new, disease specific, health-related quality of life instrument with a well-documented validity, reliability, and responsiveness for heart failure patients in Iran. The questionnaire quantifies symptoms, physical limitations, social functioning, patient’s concept of self-efficacy, and overall quality of life. We used standard scale development methods to develop a heart failure disease-specific QoL instrument, the IHF-QoL. Factor analysis revealed a strong, clinically relevant four factor solution that shows face validity and high internal consistency reliability (Cronbach's alpha exceeded the recommended cutoff of 0.7). As noted in Table 1, items relating to symptomatology and those representing psychological and socioeconomic status have come in same group in factor analysis. This may have resulted from the concept of patients being asked about their symptoms. Patients might give their general concepts of how a symptom affects their life in their own language. Heart failure symptoms limit most aspects of social living and marital relationships. Current results seem to point out that psychosocial impact of heart failure may be more prominent in Iranian patients. Individuals vary in how badly they suffer psychologically from same level of symptoms and mostly answered the symptomatology questions by referring to psychosocial-economic states. In contrast, questions assessing the daily activities with included clear guidance (drawings) and therefore pointing out to the limitations more precisely, were less affected by patient’s psychosocial state.

In our rough experience, we’ve been dealing with many heart failure patients complaining from palpitations and GI symptoms that warrants assessment of these symptoms in adjunct to well-known heart failure symptoms (dyspnea, edema, etc.) in IHF-QoL. Factor analysis clearly separated palpitation and GI symptoms from other symptoms, indicating that these symptoms might be affected by comorbidities and medications and not solely by heart failure. Finally it is worthy to mention that, to our knowledge, this is the first quality of life questionnaire being validated in patients suffering from not systolic heart failure including diastolic or right sided heart failures. In conclusion, the preliminary results of IHF-QOL questionnaire seem to indicate that it can be applied in daily clinical practice and in the clinical research context for Iranian patients with heart failure.
